# Role of Hyaluronic Acid in Post-Blepharoplasty Volume Restoration and Complication Management: A Systematic Review

**DOI:** 10.3390/jcm14134572

**Published:** 2025-06-27

**Authors:** Alaa Safia, Uday Abd Elhadi, Shlomo Merchavy, Ramzy Batheesh, Naji Bathish

**Affiliations:** 1 Department of Otolaryngology, Rebecca Ziv Medical Center, Safed 1311001, Israel; udayabdi510@gmail.com (U.A.E.); shlomo.m@ziv.health.gov.il (S.M.); 2 Department of Dermatology, Rambam Health Care Campus, Haifa 3100000, Israel; ramzy_ra512@hotmail.com; 3 Department of Dermatology, Rebecca Ziv Medical Center, Safed 1311001, Israel; najibathish@gmail.com

**Keywords:** hyaluronic acid, blepharoplasty, volume restoration, hyaluronidase, post-surgical complications, esthetic outcomes, periocular rejuvenation, revision surgery, lipofilling

## Abstract

**Background:** Hyaluronic acid (HA) has emerged as a favored adjunct to restore volume after blepharoplasty and is very effective in the treatment of postoperative hollowness, sagging, and asymmetry. Its efficacy, rate of complications, and optimal injection technique are different in different clinical studies. Hyaluronidase has been studied by diverse methods in the treatment of HA complications, including chronic edema and surgical distortion. This study critically evaluated the efficacy, safety, and technical aspects of HA in the context of blepharoplasty outcomes. **Methods:** A systematic review was performed to evaluate the use of HA and hyaluronidase for post-blepharoplasty volume rejuvenation and the treatment of complications. Studies describing HA injection technique, time interval between blepharoplasty and injection, volumetric maintenance, complication rates, esthetic and functional results, and patient satisfaction scores were considered. Risk of bias was estimated with the ROBINS-I tool. **Results:** Sample sizes across the five included studies ranged from 5 to 109 patients, and follow-up intervals ranged from 1 month to 7 years. The age of patients ranged from 31 to 76 years, and females accounted for 86% of the participants in some studies. Injection of HA successfully restored meaningful volume, with retention persisting for over 12 months in the majority of cases. HA preoperative injection caused significant patient satisfaction in a short duration and was not associated with severe complications; delayed injection caused slight distortions in some revision operations. Lipofilling showed a reduced rate of complications (12%) compared with isolated blepharoplasty (20%), suggesting its utility as an adjuvant procedure of volume restoration. Hyaluronidase successfully treated recalcitrant edema, with improvements ranging from 50% to 100%, while the application of adjuvant RF microneedling caused complete remission (100%) in subjects with multiple treatments. The application of ultrasound imaging made measurements more precise, although methods of clinical assessment were significantly heterogeneous among the studies. **Conclusions:** HA displayed efficacy in terms of efficient volume restoration after blepharoplasty, especially when technique, time, and filler selection are meticulously optimized. In comparison to lipofilling, HA is seen as somewhat safer because of its reversibility and lower likelihood of adverse vascular events. Nonetheless, considerable variability in filler type, amount, timing of administration, and result evaluation constrains conclusive clinical recommendations. The use of hyaluronidase is an effective remedial approach for overcorrection or ongoing edema.

## 1. Introduction

Blepharoplasty is among the most frequent periocular rejuvenation procedures designed to restore a youthful and rested appearance by removing excess skin, repositioning or excising orbital fat, and addressing laxity of the underlying musculature [1]. Whereas the final goal of the procedure is to enhance cosmetic outcomes and, for certain patients, maximize visual acuity by removing obstructions formed by dermatochalasis, the literature increasingly foregrounds post-blepharoplasty volume loss as a primary and often overlooked concern [2].

This loss of volume may be due to excessively aggressive removal of fat, periorbital thinning with increasing age, or progressive remodeling of the skeleton, leading to a hollow, unnatural appearance of the face that paradoxically contributes to an aged look despite surgery [3]. These sequelae after surgery are most apparent in patients with preoperative periorbital thinning, transconjunctival fat removal of the lower eyelid, and in those with inadequate tissue redistribution following surgery [4]. The progressive loss of periorbital fat, superimposed on intrinsic and extrinsic aging mechanisms, underlines the necessity for volume-preserving or volume-restoring techniques to complement standard blepharoplasty treatments [5].

Hyaluronic acid (HA) fillers have gained increased popularity as a minimally invasive, reversible, and effective post-blepharoplasty volume restorative technique, particularly in cases where soft-tissue deficiency leads to esthetic dissatisfaction [6]. HA is a glycosaminoglycan present naturally in the extracellular matrix with a high water-binding capacity and viscoelasticity that makes it an ideal candidate for soft tissue augmentation and hydration [7].

The special rheological characteristics of HA, i.e., cohesivity, elasticity, and enzyme resistance, make it effective in reestablishing periorbital contours with a low risk of overcorrection or migration [8]. Moreover, HA has been shown to influence fibroblast activity and collagen remodeling, which has the potential to influence optimizing postoperative healing and tissue integration following blepharoplasty [9].

HA’s role in the periorbital region is more than volume replacement, as its application has been associated with improved skin elasticity, hydration, and dermal remodeling, potentially reducing post-blepharoplasty textural changes and hollowness [10]. However, despite its increasing application in clinical practice, evidence for the systematic use of HA following blepharoplasty remains piecemeal, with significant heterogeneity for product selection, method of injection, timing of injection, and measurement of outcome [11].

While HA injections are a fast and minimally invasive remedy for hollowness following blepharoplasty, concerns in terms of procedural safety, durability of the correction, and complications—e.g., the Tyndall effect, nodularity, delayed hypersensitivity reaction, and, in the most unlikely of cases, vascular occlusion—require a mechanized and evidence-based course of its use in this case.

This systematic review, therefore, aimed to synthesize evidence regarding the safety and effectiveness of HA in restoring volume following blepharoplasty, its usefulness in improving patient satisfaction and reducing the need for revision surgical interventions, and establishing clinical guidelines for application in this manner.

## 2. Materials and Methods

### 2.1. Eligibility Criteria

The PECOS protocol was developed for this systematic review according to the PRISMA 2020 (Preferred Reporting Items for Systematic Reviews and Meta-Analyses) 2020 guidelines [12] to achieve reproducibility and transparency in data selection, collection, and synthesis. Population (P) included adult patients undergoing upper or lower blepharoplasty, regardless of technique, who subsequently had HA injections as volume enhancers. The Exposure (E) consisted of postoperative HA filler administration, varying from injection technique, product, and timing of filler injection. The Comparator (C) was subjects who were administered no filler after blepharoplasty or other volume augmentation procedures, such as autologous fat grafts or secondary revisions. The Outcomes (O) were patient satisfaction scores, validated esthetic rating scales, complication rates (e.g., Tyndall effect, nodularity, vascular compromise), durability of volume correction, and the need for revision procedures. The Study design (S) was randomized controlled trials (RCTs), prospective and retrospective cohort studies, case series, and case–control studies.

### 2.2. Inclusion and Exclusion Criteria

Trials that were included would have met the following requirements: possessed at least one quantitative outcome in terms of patient satisfaction, complication, or maintenance of volume, and represented a comparative or observational study design with an identifiable methodology. Articles published in English and peer-reviewed journals were considered to ensure methodological rigor.

Case reports, narrative reviews, expert opinions, letters to the editor, and conference abstracts with no primary data were excluded. Studies comparing HA use in non-surgical periocular rejuvenation or in patients with underlying medical diseases affecting wound healing (e.g., autoimmune diseases, connective tissue disorders) were excluded to restrict confounding factors. Additionally, articles with limited statistical analysis (studies that lacked sufficient rigor in their statistical methodology to support valid or generalizable conclusions), no control, or replicate data from published studies were excluded from the final synthesis.

### 2.3. Database Search Protocol

We carried out an extensive literature search of PubMed, Embase, Scopus, Web of Science, Cochrane Library, CINAHL, and LILACS to find studies that evaluated the application of HA in post-blepharoplasty patients. The search strategy was constructed on the basis of a combination of MeSH terms, Boolean operators, and free-text words to guarantee high sensitivity as well as specificity. Truncation and wildcards were utilized to include spelling differences. Boolean operators “AND,” “OR,” and “NOT” were systematically applied to narrow down the search queries and to remove undesirable studies. The search was restricted to human studies only, without any date limit to enable a full historical overview of the topic. Search terms were tailored to each database to fit its indexing style. The final search result was exported into EndNote, and duplicates were removed before screening. There were no limitations to the time period of the search protocol, with the search date ranging from inception till March 2025 (Table 1).

### 2.4. Data Extraction Protocol and Data Items

Data extraction was performed in a systematic way using a pre-formatted extraction sheet to ensure consistency and accuracy. The data extracted included study characteristics (author, year, country, study design, sample size), patient factors (age, sex, comorbidities), surgical factors (type of blepharoplasty, fat preservation/removal technique, adjunctive procedures), HA treatment characteristics (type of filler, injection technique, volume used, timing of use), comparators (control groups, other methods of volume restoration), outcome measures (patient satisfaction scores, validated assessment scales, objective measures of volume retention, rate of complications), and follow-up duration. Other criteria included the statistical methodologies applied and sources of funding to identify any conflicts of interest. Data was retrieved separately by two reviewers, and disagreements were addressed by a third reviewer to minimize bias.

The literature search was extensive and covered seven major databases, including PubMed, Embase, Scopus, Web of Science, Cochrane Library, CINAHL, and LILACS. Despite a broad search strategy without date restrictions, only five clinical studies ultimately fulfilled the inclusion criteria. This was due to strict methodological filters applied to study design, outcome relevance, and exclusion of non-clinical or insufficiently detailed reports. The final selection was based on PECOS guidelines, focusing on human subjects undergoing blepharoplasty followed by HA intervention. Given the heterogeneity in methodology and outcome measures, statistical synthesis was not conducted, and results were synthesized qualitatively. All references to statistical pooling or numerical effect sizes were removed, as no meta-analysis was feasible due to substantial variability among included studies.

### 2.5. Bias Assessment Protocol

Risk of bias was assessed using the ROBINS-I (Risk of Bias in Non-Randomized Studies of Interventions) instrument [13] for observational studies and Cochrane’s RoB 2.0 tool [14] for randomized trials. The ROBINS-I tool measured potential biases in confounding, selection of participants, classification of intervention, deviations from the intended intervention, missing data, measuring outcomes, and selective reporting. The trials were categorized as low, moderate, serious, or critical risk of bias. The Cochrane RoB 2.0 tool reviewed sequence generation, allocation concealment, blinding, incomplete outcome data, and selective reporting. Each of these areas was scored as having low, some concern, or high levels of risk of bias. Differences in the evaluation of bias were managed through discussion among reviewers to achieve an objective assessment.

## 3. Results

**Figure 1:**
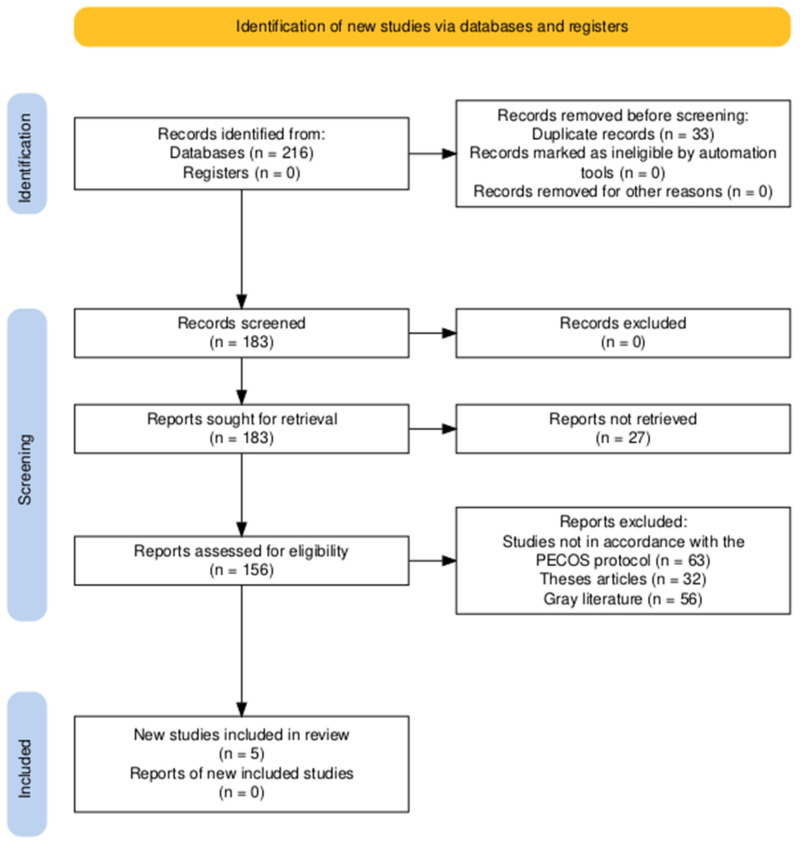
PRISMA study selection process for the review.

In total, 216 records were found from database searching, and no records were found from registers (Figure 1). Before screening, 33 records were excluded because of duplication, and no records were excluded because of automation tools or other reasons. After deduplication, 183 records were screened, and no records were excluded at this stage. All 183 reports were requested for retrieval, of which 27 reports were not retrieved. Thus, 156 reports were screened for study eligibility. Out of these, 151 studies were excluded based on several criteria, including non-compliance with the PECOS protocol (n = 63), categorization as thesis articles (n = 32), and categorization as gray literature (n = 56). Five studies [15,16,17,18,19] ultimately met the inclusion criteria and were included in the systematic review.

### 3.1. Population Distribution

The included studies were conducted over a span of a decade (Table 2), from 2015 [19] to 2024 [15,16], across studies in Italy [15,19] and the USA [16,17,18]. The research designs varied, including retrospective studies [15,16,18], a case series [17], and a prospective cohort study [19], offering different research methodologies for the evaluation of HA outcomes. The sample sizes varied greatly, ranging from as few as five cases in the targeted case series [17] to as many as 109 patients in a large retrospective study [15]. The participants’ age varied by mean, from 31 to 76 years [16], offering a wide demographic sample, while one study had a smaller age range of 47 (28–68) years [18]. Female patients outnumbered the population in all the studies, with up to 86% in one cohort [15] and entirely female populations in another [17]. The follow-up times ranged from brief monitoring of 1 month [19] to long-term observational studies for up to 7 years [16], offering both short-term and long-term outcome measurements.

### 3.2. Application and Methods of Injecting Hyaluronic Acid

The investigations differed regarding the type of HA employed (Table 3), with some being particular to cross-linked HA gel (brand unspecified) [18], while others contrasted named fillers like Juvederm, Restylane, Voluma, and Sculptra [17]. One investigation contrasted only lipofilling with HA injection [15], while another contrasted the impact of high-dose hyaluronidase on HA-induced complications of the lower eyelid [16]. The timing of injection varied widely, with investigations employing transconjunctival high-dose hyaluronidase [16], percutaneous hyaluronidase and radiofrequency (RF) microneedling [17], and multi-plane cannula injections (25G) [19]. The timing of HA injection in relation to blepharoplasty varied widely, with the immediate pre-surgical administration in some investigations [19], as opposed to investigations where HA had been injected years earlier before surgical treatment was commenced [18]. The dose of HA injected was accurately recorded in one study, with an average of 0.8 mL per side [19], while other investigations varied injected volumes from 30 to 75 units of Hylenex per session [17] or did not provide the exact volume utilized [18].

### 3.3. Complication Rates and Imaging Evaluations

Complication rates differed dramatically by treatment modality. A study reported increased rates of complications in the group undergoing blepharoplasty alone (20%) in comparison to combined blepharoplasty with lipofilling (12%) [15], suggesting a possibly protective effect of lipofilling in reducing complications. There were no severe complications reported in high-dose hyaluronidase [16] and serial hyaluronidase plus RF microneedling [17] studies; thus, there is a low risk of injury with these procedures. A study, however, revealed minor surgically related anomalies in those patients previously treated with HA followed by blepharoplasty [18], suggesting potential surgical technique complications in eyelids treated with HA. Study designs for imaging and assessment were varied, including subjective pain score and patient satisfaction questionnaires [15], ultrasound and digital photography grading [17], surgical evaluations by a blinded observer [18], and brow height observations at 1 week and 1 month follow-up [19].

### 3.4. Clinical Effectiveness and Patient Outcomes

Clinical effect duration demonstrated different findings between studies (Table 4). One revealed long-term effects beyond 12 months [19], while the other demonstrated extensive improvements that sustained for a period of at least 24 months without recurrence [17]. One other study identified long-term gains but added that the aging process continued to have an impact over time [15]. The esthetic and functional results were recorded as positive in all studies, some recording complete recovery from complications in two cases and resolution using multiple treatments in three cases [17]. Another study, however, suggested that some of the patients had post-surgical filler adjustments that needed to be made to achieve maximum esthetic improvement [18]. One study showed considerable brow height restoration improvements, while high levels of patient satisfaction in the intervention arm were observed as well [19]. In the resolution of edema, one reported a 50–100% improvement rate post-treatment using high-dose hyaluronidase and no recurrence noted [16].

### 3.5. Post-Injection Management

Post-procedure management techniques were also diverse, with some employing cold compresses and NSAIDs to alleviate symptoms [19], whereas others employed hyaluronidase as well as surgical contouring of the fat for increased esthetic enhancement [16]. Adjuvant modalities like RF microneedling and CO_2_ resurfacing were employed in some instances to enhance skin texture and reduce volume loss [17]. Overall conclusions confirmed the efficacy of HA-based therapy in the post-blepharoplasty volume restoration, with few complications noted. One mentioned the advantage of lipofilling in optimizing the results of blepharoplasty with decreased complication rates post-operation [15]. Another mentioned the advantage of high-dose hyaluronidase in treating chronic lower eyelid edema after previous HA injections [16]. Additionally, serial hyaluronidase with RF microneedling was found to be an effective modality in the treatment of post-hyaluronic acid recurrent eyelid edema (PHAREE) [17]. Although revision blepharoplasty in HA-treated eyelids was believed to be feasible, it was a surgical challenge, and careful patient selection and planning were required [18].

The findings from the included studies suggested a potential role for HA in improving post-blepharoplasty volume outcomes and patient satisfaction. However, the strength of these findings was constrained by the methodological limitations of the studies included. Most studies employed retrospective or small-scale designs with subjective outcome measures and variable follow-up durations. The use of different HA formulations, injection planes, and timing further introduced variability, limiting the generalizability of results. Safety outcomes were inconsistently reported, and claims of efficacy varied widely due to non-standardized assessment tools. While hyaluronidase was reported to reduce edema and correct complications effectively, treatment protocols differed across studies, making definitive comparisons difficult. The lack of objective volumetric assessment tools and consistent complication grading systems highlighted the need for standardized methodologies. Future research should focus on prospective trials with unified outcome measures and longer follow-up durations to guide clinical protocols more reliably.

### 3.6. Bias Assessment Observations

**Figure 2:**
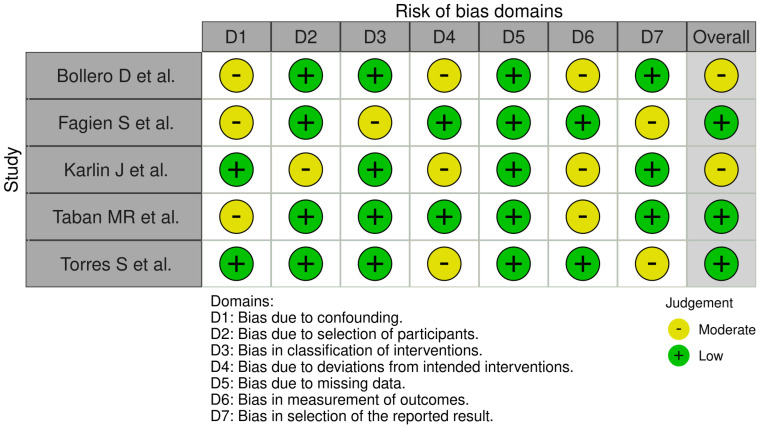
Bias assessment observations [15,16,17,18,19].

Bollero et al. [15] had a moderate risk of bias overall, primarily due to problems with confounding (D1), missing data (D4), and selection bias in reporting (D6), with low risk otherwise (Figure 2). Fagien et al. [16] had overall low risk of bias, but only moderate risk in confounding (D1) and measurement of outcomes (D7), and only minimal problems in these areas. Karlin et al. [17] had an overall moderate risk but moderate risk in deviations from intended interventions (D2), missing data (D4), and selection bias in reporting (D6), but low risk otherwise. Taban et al. [18] had an overall low risk, but only moderate risk in confounding (D1) and selection bias in reporting (D6), with low risk otherwise. Torres et al. [19] had an overall low risk of bias as well, but only deviations from intended interventions (D4) and measurement of outcomes (D7) posed a moderate risk, with low risk otherwise. Overall, most of the studies had low to moderate risk of bias, with heterogeneity primarily in confounding, deviations from interventions, and selection bias in reporting, but overall methodological soundness with only minor problems in individual aspects.

## 4. Discussion

### 4.1. Comparative Analysis of Findings

The final inclusion of only five studies despite an extensive, multi-database search was primarily due to the limited availability of clinical investigations that met rigorous PECOS-based eligibility criteria. Many excluded studies lacked methodological robustness, did not focus specifically on postoperative HA use in blepharoplasty, or failed to report meaningful clinical outcomes. This narrow evidence base reflects the nascent and heterogeneous nature of research in this domain.

The findings across the included studies demonstrated some thematic overlaps but also revealed significant methodological and clinical variability. For example, Bollero et al. [15] compared standalone blepharoplasty to its combination with lipofilling, while Torres et al. [19] and Taban et al. [18] directly assessed HA use in volume restoration. While reduced complication rates were noted in lipofilling groups in Bollero et al. [15] and minimal adverse events were reported in HA-treated patients in Torres et al. [19], these outcomes should be interpreted cautiously. Taban et al. [18] observed minor surgical distortions in patients with long-standing prior HA injections, highlighting the potential complexities associated with delayed filler degradation and tissue interaction. However, small sample sizes, subjective outcome measures, and inconsistent reporting protocols limit the strength of these observations.

Studies investigating hyaluronidase for complication management—Fagien et al. [16] and Karlin et al. [17]—reported improvements in edema and soft tissue irregularities following various treatment protocols. However, variations in injection techniques, dosing, and adjunctive therapies such as RF microneedling confound direct comparison. Although reported improvements ranged from partial to complete resolution, the absence of standardized measurement tools and small case series design precludes generalization. Notably, only Karlin et al. [17] employed objective imaging for volume assessment, whereas others relied on subjective scales, further emphasizing the need for consistent methodology. Follow-up periods also differed widely, ranging from short-term assessments to multi-year observations, complicating the interpretation of long-term outcomes.

While the review briefly highlights adjunctive surgical procedures such as canthopexy or canthoplasty, their specific interactions with HA outcomes remain underexplored in the existing literature. These techniques, although well-established for correcting anatomical laxity, were not quantitatively assessed in any included study in terms of their impact on postoperative HA behavior or complication profiles.

Similarly, discussions around lower eyelid blepharoplasty, dry eye syndrome, and blinking dysfunction underscore important clinical considerations but were not central endpoints in the studies reviewed. While these complications are well-documented in broader oculoplastic literature, none of the included studies directly assessed the influence of HA or hyaluronidase on these parameters using objective metrics. Therefore, drawing associations between HA use and postoperative ocular surface complications remains speculative based on current evidence.

### 4.2. Contextual Considerations

Although not systematically evaluated in the included studies, several clinically relevant considerations—such as adjunctive procedures like canthopexy and canthoplasty, postoperative dry eye, blinking dysfunction, and eyelid malposition—warrant brief narrative mention due to their impact on surgical outcomes. Canthopexy is typically used for minimal lateral canthal laxity, involving fixation of the lateral canthal tendon to the orbital rim [4], while canthoplasty is reserved for more severe laxity, allowing for tendon repositioning and improved eyelid support [8,19,20,21,22,23]. Lower eyelid blepharoplasty poses greater biomechanical challenges than upper eyelid surgery due to its need to counter gravity, often leading to complications like ectropion or lid retraction when support is inadequate [24,25]. Postoperative dry eye, reported in up to 26.5% of cases, is more frequent in combined upper and lower lid procedures and is often exacerbated by eyelid descent, incomplete blinking, or excessive tissue resection [23,24,25,26].

### 4.3. Blinking Dysfunction and Tear Film Instability

Besides eyelid malposition, impairment of the blink mechanism postoperatively can produce instability of the tear film [27,28,29]. A decreased blink frequency, in turn, is likely to result from surgical reorganization of the orbicularis oculi muscle and can cause increased evaporation of tear loss, leading to exacerbation of ocular surface dryness. The tear distribution compromise due to blepharoplasty can also further jeopardize ocular lubrication as well as make preoperative symptoms of dryness worse in prone individuals [1,30,31,32,33].

### 4.4. Management Strategies for Dry Eye Post-Surgery

Alleviation of inflammation, restoration of the stability of the tear film, and tissue healing are the strategies taken in the approach to postoperative dry eye. Conservative management encompasses elevation of the head to attenuate edema, the use of cold compresses, and the utilization of lubricating eye drops, as well as application of topical corticosteroids and antibiotics with the aim to minimize infection potential [27]. Most cases follow a three-month course; otherwise, chronic complaints may require resorting to further modalities. For example, punctal occlusion to maximize tear retention, or repositioning operations to restore lid positioning and maintain tear function [27].

### 4.5. Limitations of the Review

The results derived from the studies were also plagued by numerous methodological and clinical limitations, which impacted their generalizability to larger patient populations. A primary limitation reported was heterogeneity in study designs, the studies by Bollero et al. [15], Fagien et al. [16], and Taban et al. [18] being predominantly retrospective, hence naturally prone to selection bias, inadequate documentation of patient backgrounds, and inconsistency in data collection protocols. The addition of a case series by Karlin et al. [17] also diminished statistical power, as small sample sizes compromised extrapolation ability. Additionally, the follow-up period also exhibited broad variability across the studies, with the follow-up periods ranging from as little as 1 month in Torres et al. [19] to over 7 years in Fagien et al. [16], hence preventing uniform evaluation of long-term efficacy and rates of complications.

Differences in the rheological properties of HA products, such as Juvederm, Restylane, and Voluma, were not critically correlated with either volumization efficacy or complication rates. These fillers differ in cohesivity and G’ (elastic modulus), which may influence their propensity to migrate or cause contour irregularities. Additionally, the plane of injection (deep vs. superficial) and delivery tool (needle vs. cannula) were inconsistently reported and rarely linked to clinical outcomes. For instance, cannula use—generally associated with lower vascular injury risk—was not specifically assessed for its complication profile compared to needle-based injections.

Moreover, variations in the timing of HA administration substantially impacted outcome interpretation. Torres et al. [19] utilized immediate pre-surgical HA injection, whereas Taban et al. [18] included patients with historical filler use, often years prior to surgery, limiting comparability. The volume injected was poorly documented across studies, with only Torres et al. [19] reporting a mean of 0.8 mL per side. This lack of standardization precludes meaningful comparisons of volume retention and complication rates.

Furthermore, while objective imaging methods such as ultrasound and photographic grading were employed by Karlin et al. [17], most studies relied on subjective satisfaction scores or non-standardized clinical assessments [15,18], which compromised inter-study comparability. The underutilization of imaging technology in outcome assessment remains a missed opportunity.

### 4.6. Clinical Recommendations

To improve the consistency and safety of HA-based volume restoration post-blepharoplasty, future research and clinical practice should consider the following:

- **Optimal timing**: Delayed postoperative HA injection may reduce complication risks such as edema compared to immediate or preoperative administration [19].
- **HA vs. lipofilling**: HA demonstrates a more favorable safety profile due to reversibility and lower risk of vascular compromise, making it a preferable option in high-risk anatomical zones [15,16,17,18].
- **Use of hyaluronidase**: Hyaluronidase should be used as first-line treatment in cases of overcorrection, persistent edema, or suspected vascular compromise related to HA injection.
- **Injection technique**: Deep-plane injections via blunt cannula may minimize trauma and allow more controlled filler placement.
- **Outcome assessment**: Standardization of measurement tools, particularly ultrasound and photographic volumetric grading, is critical to objectively evaluate efficacy and complications.
- **Protocol standardization**: Future trials should adopt consistent protocols regarding HA type, injection volume, technique, and timing to enable valid meta-analyses.

## 5. Conclusions

HA demonstrated potential for effective post-blepharoplasty volume restoration, particularly when technique, timing, and filler selection are optimized. Compared to lipofilling, HA appears relatively safer, owing to its reversibility and lower risk of adverse vascular events. However, substantial heterogeneity in filler type, volume, timing of administration, and outcome assessment limits definitive clinical recommendations. The use of hyaluronidase provides a valuable corrective strategy in the event of overcorrection or persistent edema. To improve consistency and safety, future research should prioritize the standardization of injection techniques, incorporate objective tools such as ultrasound, and include extended follow-up with complication grading systems. Only through such rigor can reliable clinical guidelines be formulated.

## Figures and Tables

**Table 1:** Database search strings.

Database	Search String
**PubMed**	(“Blepharoplasty”[MeSH] OR “Eyelid Surgery”[MeSH] OR “Periorbital Surgery”) AND (“Hyaluronic Acid”[MeSH] OR “Hyaluronic Acid Filler” OR “HA Injection” OR “Soft Tissue Augmentation”) AND (“Volume Restoration” OR “Periorbital Hollowness” OR “Tear Trough Correction”) AND (“Outcomes” OR “Complications” OR “Patient Satisfaction”) NOT (“Animal Studies”)
**Embase**	(‘blepharoplasty’/exp OR ‘eyelid surgery’/exp OR ‘periorbital rejuvenation’) AND (‘hyaluronic acid’/exp OR ‘dermal filler’/exp OR ‘tissue augmentation’) AND (‘aesthetic outcome’ OR ‘complication’/exp OR ‘satisfaction’/exp) AND [humans]/lim
**Scopus**	TITLE-ABS-KEY (“Blepharoplasty” OR “Eyelid Surgery”) AND TITLE-ABS-KEY (“Hyaluronic Acid” OR “Soft Tissue Filler”) AND TITLE-ABS-KEY (“Volume Correction” OR “Complication Rates” OR “Patient-Reported Satisfaction”) AND NOT TITLE-ABS-KEY (“Experimental Models”)
**Web of Science**	TS = (“Blepharoplasty” OR “Eyelid Rejuvenation”) AND TS = (“Hyaluronic Acid Injection” OR “HA Filler”) AND TS = (“Volume Retention” OR “Periorbital Atrophy”) AND NOT TS = (“Preclinical Studies”)
**Cochrane Library**	(“Blepharoplasty” OR “Eyelid Surgery”) AND (“Hyaluronic Acid” OR “Dermal Fillers”) AND (“Volume Restoration” OR “Long-Term Outcomes”) in Title, Abstract, Keywords
**CINAHL**	(MH “Blepharoplasty”) AND (MH “Hyaluronic Acid”) AND (“Volume Augmentation” OR “Aesthetic Outcomes”) AND (“Complication Prevention”) NOT (“Non-Human Studies”)
**LILACS**	(Blefaroplastia OR Cirugía de párpados) AND (Ácido hialurónico OR Inyección dérmica) AND (Restauración de volumen OR Oclusión vascular) NOT (Estudios en animales)

**Table 2:** Demographic characteristics of the studies included in the review.

Author ID	Year	Location	Study Design	Sample Size	Mean Age (in Years)	Male:Female Ratio	Follow-up Period
**Bollero D et al. [15]**	2024	Italy, USA	Retrospective Study	109	54.2 ± 10.1	86% Females	Short-term (not specified)
**Fagien S et al. [16]**	2024	USA	Retrospective Cohort	70	31–76	0.253472222	1 month to 7 years
**Karlin J et al. [17]**	2023	USA	Case Series	5	49–64 (Mean 55.4)	All females	24–28 months
**Taban MR et al. [18]**	2017	USA	Retrospective Study	15	47 (28–68)	02:13	6 months to 2 years
**Torres S et al. [19]**	2015	Italy	Prospective Cohort	15	42–69	05:10	1 month

**Table 3:** Injection parameters and clinical application.

Author ID	Groups Assessed	Type of Hyaluronic Acid Used	Injection Technique	Time Interval Between Blepharoplasty and HA Injection	Volume of HA Injected
**Bollero D et al. [15]**	Blepharoplasty vs. Blepharoplasty + Lipofilling	Not applicable (lipofilling)	Not applicable	Not applicable	Not applicable
**Fagien S et al. [16]**	Lower blepharoplasty + High-dose hyaluronidase	Various HA fillers (patient history unclear)	Transconjunctival high-dose hyaluronidase	Varied (weeks to >10 years post HA injection)	Not clearly reported
**Karlin J et al. [17]**	Serial hyaluronidase + RF microneedling	Juvederm, Restylane, Voluma, Autologous Fat, Sculptra	Percutaneous hyaluronidase + RF microneedling	Varied (multiple filler injections over the years)	30–75 units of Hylenex/session
**Taban MR et al. [18]**	Blepharoplasty in HA-treated eyelids	Cross-linked HA gel (brand not specified)	Prior HA injections varied, some received hyaluronidase	Varied (some patients had prior HA injection years ago)	Not specified
**Torres S et al. [19]**	HA + Blepharoplasty vs. Control	Intraline (cross-linked, spherification tech)	Cannula (25G, multi-plane)	Immediate pre-surgery	0.8 mL per side

**Table 4:** Outcomes, complications, and management.

Author ID	Complication Rates and Types	Imaging or Objective Assessment Method	Duration of Clinical Effect	Functional and Esthetic Outcomes	Post-Injection Management and Adjunctive Therapies	Conclusion Assessed
**Bollero D et al. [15]**	Higher in blepharoplasty alone (20%) vs. combined (12%)	Pain score, patient satisfaction scales	Long-term benefit, but aging continues	Higher satisfaction in the lipofilling group	NSAIDs, PSWT device for pain/inflammation	Lipofilling enhances blepharoplasty outcomes with minimal added risks
**Fagien S et al. [16]**	No major complications, persistent edema resolved	Clinical observation, patient-reported outcomes	Varied, 50–100% improvement reported	Significant edema reduction, improved esthetics	NSAIDs, hyaluronidase, and surgical fat contouring	High-dose hyaluronidase effectively treats chronic post-HA edema
**Karlin J et al. [17]**	No adverse effects, no recurrence	Ultrasound assessment, photographic grading	Minimum 24 months without recurrence	Complete resolution in 2 cases, multi-session in 3 cases	RF microneedling, CO_2_ laser in select patients	Serial hyaluronidase + RF microneedling is effective for PHAREE
**Taban MR et al. [18]**	No major complications, surgical distortion noted	Blind observer assessment, surgical evaluation	Not clearly stated, some required ‘touch-up’	All satisfied; some required post-surgical filler	Hyaluronidase used pre- or intra-operatively	Revision is feasible but challenging in HA-treated eyelids
**Torres S et al. [19]**	None reported	Brow height measurement (1 week, 1 month)	>12 months	Significant brow height improvement, high satisfaction	Cold compress, NSAIDs	HA provides effective volume restoration without added complications

## Data Availability

No new data were created or analyzed in this study.

## References

[1] Liu X., Gao Y., Ma J., Li J. (2024). The Efficacy and Safety of Hyaluronic Acid Injection in Tear Trough Deformity: A Systematic Review and Meta-analysis. Aesthetic Plast. Surg..

[2] Woodward J., Cox S.E., Kato K., Urdiales-Galvez F., Boyd C., Ashourian N. (2023). Infraorbital Hollow Rejuvenation: Considerations, Complications, and the Contributions of Midface Volumization. Aesthet. Surg. J. Open Forum..

[3] Tabassum N., Chowdary Jasthi V., Al Salem A., Kumar S.M., Muayad Alshaban M., Alrashd D.M., Al Nasser L., Ahmed S. (2023). Perspectives and challenges in lip rejuvenation: A systematic review. Eur. Rev. Med. Pharmacol. Sci..

[4] de Sousa A.M.S., Duarte A.C., Decnop M., Guimarães D.F., Coelho Neto C.A.F., Sarpi M.O., Duarte L.G.P., Souza S.A., Segato L.F., Zavariz J.D. (2023). Imaging Features and Complications of Facial Cosmetic Procedures. Radiographics.

[5] Maione L., Vinci V., Costanzo D., Battistini A., Lisa A., Di Maria A. (2021). Upper eyelid blepharoplasty following hyaluronic acid injection with improved facial aesthetics and eye symptoms: A case report. J. Med. Case Rep..

[6] Miotti G., Zeppieri M., Pederzani G., Salati C., Parodi P.C. (2023). Modern blepharoplasty: From bench to bedside. World J. Clin. Cases.

[7] Diaspro A., Calvisi L., Sito G. (2022). Hyaluronic Acid Gel Injection for the Treatment of Tear Trough Deformity: A Multicenter, Observational, Single-Blind Study. Aesthetic Plast. Surg..

[8] Lamkin I., Pugliese B., Nystrom J., Fubini S.L., Knickelbein K.E. (2024). Restoration of function following traumatic superior eyelid avulsion in a horse treated with advancement flap blepharoplasty (H-plasty) and subdermal hyaluronic acid filler. Vet. Ophthalmol..

[9] Sawant O., Khan T. (2020). Management of periorbital hyperpigmentation: An overview of nature-based agents and alternative approaches. Dermatol. Ther..

[10] Alharbi M.M., Bin Dlaim M.S., Alqahtani J.M., Alkhudhairy N.S., Almasoudi S.M., Alajmi N.T. (2023). Ophthalmic Complications of Periorbital and Facial Aesthetic Procedures: A Literature Review. Cureus.

[11] Barone M., Salzillo R., Persichetti P. (2022). Role of the Fill and Lift Effect with Hyaluronic Acid on the Middle Third of the Face: What Are the Effects on the Lower Eyelid?. Aesthetic Plast. Surg..

[12] Page M.J., Moher D., Bossuyt P.M., Boutron I., Hoffmann T.C., Mulrow C.D., Shamseer L., Tetzlaff J.M., Akl E.A., Brennan S.E. (2021). PRISMA 2020 explanation and elaboration: Updated guidance and exemplars for reporting systematic reviews. BMJ.

[13] Igelström E., Campbell M., Craig P., Katikireddi S.V. (2021). Cochrane’s risk of bias tool for non-randomized studies (ROBINS-I) is frequently misapplied: A methodological systematic review. J. Clin. Epidemiol..

[14] Sterne J.A.C., Savović J., Page M.J., Elbers R.G., Blencowe N.S., Boutron I., Cates C.J., Cheng H.Y., Corbett M.S., Eldridge S.M. (2019). RoB 2: A revised tool for assessing risk of bias in randomised trials. BMJ.

[15] Bollero D., Donzelli S., Bovani B., Tretti Clementoni M., Melfa F., Piccolo D., Keener M. (2024). Integration of Lipofilling with Blepharoplasty to Optimize Periorbital Rejuvenation: A Retrospective Study. J. Ski. Stem Cell.

[16] Fagien S. (2024). The Treatment of Cosmetic Lower Eyelid Adverse Events After Injection of Hyaluronic Acid Gel Fillers. Aesthet. Surg. J. Open Forum..

[17] Karlin J., Vranis N., Dayan E., Parsa K. (2023). Post-Hyaluronic Acid Recurrent Eyelid Edema: Pathophysiologic Mechanisms and a Proposed Treatment Protocol. Aesthet. Surg. J. Open Forum..

[18] Taban M.R. (2017). Lower Blepharoplasty in Eyelids Previously Injected with Hyaluronic Acid Gel Filler. Am. J. Cosmet. Surg..

[19] Torres S. (2015). Volumetric Eyebrow Lifting with the Aid of a New Hyaluronic Acid Dermal Filler (Intraline) and Upper Surgical Blepharoplasty; Enhancing Outcomes. J. Clin. Exp. Dermatol. Res..

[20] Duque Clavijo V., Suarez S., Espinosa-Reyes J., Gonzalez C., Rolom M., Sinuco Rueda L. (2024). When Hyaluronic Acid Alters its Rheology: Clinical, Ultrasonographic and Pathological Correlation. J. Adv. Plast. Surg. Res..

[21] Bhattacharjee K., Ghosh S., Ugradar S., Azhdam A.M. (2020). Lower eyelid blepharoplasty: An overview. Indian J. Ophthalmol..

[22] Meihofer A., Foy V., Zambrano R., Rozenberg S. (2025). Keratoconjunctivitis Sicca Due to Blepharoplasty Treated with Neurotoxin and Hyaluronic Acid Filler. Am. J. Cosmet. Surg..

[23] Fan W., Rokohl A.C., Guo Y., Heindl L.M. (2021). Ocular surface and tear film changes after eyelid surgery. Ann. Eye Sci..

[24] Labib A., Patel B.C., Milroy C. (2024). Blepharoplasty, Lower Lid, Canthal Support.

[25] Rostami S., de la Torre J.I., Czyz C.N. (2023). Lower Eyelid Blepharoplasty.

[26] Kim M.J., Oh T.S. (2019). Treatment for ophthalmic paralysis: Functional and aesthetic optimization. Arch. Craniofac. Surg..

[27] Zhang S.Y., Yan Y., Fu Y. (2020). Cosmetic blepharoplasty and dry eye disease: A review of the incidence, clinical manifestations, mechanisms and prevention. Int. J. Ophthalmol..

[28] Few J., Cox S.E., Paradkar-Mitragotri D., Murphy D.K. (2015). A multicenter, single-blind randomized, controlled study of a volumizing hyaluronic acid filler for midface volume deficit: Patient-reported outcomes at 2 years. Aesthet. Surg. J..

[29] Lorenc Z.P., Corduff N., van Loghem J., Yoelin S. (2022). Creating lift in the lower face with botulinum toxin a treatment: An anatomical overview with videos and case studies illustrating patient evaluation and treatment. Aesthet. Surg. J. Open Forum..

[30] Nikolis A., Enright K.M., Berros P., Sampalis J.S. (2023). Safety of infraorbital hyaluronic acid injections: Outcomes of a meta-analysis on prospective clinical trials. J. Cosmet. Dermatol..

[31] Trinh L.N., Grond S.E., Gupta A. (2022). Dermal fillers for tear trough rejuvenation: A systematic review. Facial Plast. Surg..

[32] Colon J., Mirkin S., Hardigan P., Elias M.J., Jacobs R.J., Elias M. (2023). Adverse events reported from hyaluronic acid dermal filler injections to the facial region: A systematic review and meta-analysis. Cureus.

[33] Evans A.G., Ivanic M.G., Botros M.A., Pope R.W., Halle B.R., Glassman G.E., Genova R., Al Kassis S. (2021). Rejuvenating the periorbital area using platelet-rich plasma: A systematic review and meta-analysis. Arch. Dermatol. Res..

